# Membrane cholesterol modulates engagement of β-arrestin with the ghrelin receptor

**DOI:** 10.1038/s42003-026-09889-0

**Published:** 2026-04-01

**Authors:** Ludovic Berto, Pauline Henri, Marjorie Damian, Sonia Cantel, Jean-Alain Fehrentz, Nathalie Sibille, Michela Di Michele, Jean-Louis Banères

**Affiliations:** 1 Institut des Biomolécules Max Mousseron (IBMM), CNRS, Université de Montpellier, ENSCM, Montpellier, France; 2 Centre de Biologie Structurale (CBS), Université de Montpellier, INSERM, CNRS, Montpellier, France

**Keywords:** Biochemistry, Lipids

## Abstract

The precise contribution of lipids to G protein-coupled receptor (GPCR) signaling remains to be elucidated. Of all the lipids, cholesterol likely plays a special role due to its abundance in different membrane compartments. Here, we assembled the ghrelin receptor GHSR into lipid nanodiscs containing different amounts of cholesterol and leveraged fluorescence spectroscopy to analyze its impact on receptor conformational dynamics and ability to interact with signaling partners. We showed that specific lipid:cholesterol interactions shift the receptor conformational equilibrium toward the active/active-like states, stabilizing the complex the GHSR forms with its cognate Gq protein. In contrast, while low cholesterol levels favored arrestin recruitment through an interaction with the receptor transmembrane core, its C-terminus and the lipid bilayer, increasing the cholesterol-to-phospholipid ratio in the nanodiscs was associated with dissociation of β-arrestin1 from both the membrane and the receptor core, resulting in the formation of a tail-only engaged complex. Taken together, these data highlight the multifaceted role of membrane lipid composition as a possible modulator of the arrangement of the complex the GHSR forms with G proteins and β-arrestins, and thus as a regulator of GHSR selectivity in desensitization, endocytosis, and signaling.

## Abstract (web summary)

The authors demonstrate that the cholesterol content in the membrane may modulate the organization the ghrelin receptor forms with one of its main effectors, β-arrestin.

## Introduction

G protein-coupled receptors (GPCRs) are membrane receptors that play a critical role in many different biological processes^1^. GPCRs are conformationally dynamic proteins that explore complex energy landscapes, with multiple states responsible for the activation of downstream intracellular partners, including various G protein subtypes and β-arrestins^2^. The latter are a family of effector proteins with four members, including the two non-visual β-arrestin1 and β-arrestin2, which play a crucial role in the regulation of GPCR signaling by controlling their desensitization and internalization and by mediating intracellular signaling events^3^.

As membrane proteins, GPCRs require a suitable environment, the lipid bilayer, to maintain their native fold. However, the membrane not only provides this environment, but also modulates the function of the receptors by influencing the distribution of the different states in their energy landscape, and thereby their ability to interact with their cognate effectors^4^. This modulation results on the one hand, from the influence of the physicochemical properties of the membrane—thickness, curvature, fluidity—and on the other hand from direct interactions of the proteins with specific lipids^5^. Among all the lipids that have been shown to affect GPCR function, cholesterol has raised a particular interest because of its natural abundance and particular physicochemical properties^6^. Cholesterol is a lipid present in varying amounts in eukaryotic membranes, ranging from a few mole fractions in mitochondrial membranes to ca. 40 mol% in some domains of plasma membranes^7^. In addition, the amount of cholesterol in membranes is dynamically regulated depending on physiological and pathological conditions^7^. Cholesterol impacts on the bilayer bulk properties (thickness, fluidity) and how this affects GPCR functioning, has been particularly explored with rhodopsin^8,9^. Besides these bulk effects, three-dimensional structures have also revealed the presence of cholesterol bound at specific sites in several GPCRs^10^. Combination of computational and biophysical methods provided further evidence that such binding can regulate the functional properties of GPCRs^11^.

Among all the GPCRs that have been proposed to bind cholesterol is the ghrelin receptor. Ghrelin is a 28-amino acid gastrointestinal peptide hormone that exerts a wide range of actions, including control of growth hormone secretion, food intake, glucose metabolism, and response to reward and stress^12^. All these effects result from the interaction of ghrelin with a single GPCR, the growth hormone secretagogue receptor (GHSR), which is a promiscuous receptor activating both G protein- (Gq, Gi, G13) and β-arrestin-dependent pathways^13^. Interestingly, cholesterol molecules (or its analog cholesteryl hemisuccinate) have been reported in all the cryo-EM structures of the GHSR complexed with an agonist and a G protein^14–16^ but not in the antagonist or inverse-agonist bound crystal structures^14,17^, suggesting this lipid might preferentially bind the active state of the receptor.

Using the isolated ghrelin receptor assembled into nanodiscs of well-defined lipid composition, we analyzed here whether cholesterol influenced the conformational dynamics of the receptor and its ability to recruit signaling partners. We found that specific lipid:cholesterol interactions shifted the receptor conformational equilibrium toward the active/active-like states and stabilized the complexes the GHSR forms with G proteins. In contrast, while low cholesterol levels favored full engagement of β-arrestin1, increasing the cholesterol-to-phospholipid ratio in the nanodiscs was associated with dissociation of this signaling protein from the lipid bilayer, shifting the complex from a core to a tail-only engagement. These results point at cholesterol as an endogenous allosteric modulator of the ghrelin receptor, possibly modulating its selectivity in signaling and internalization.

## Results

### Effect of cholesterol on the GHSR conformation

**Fig. 1:**
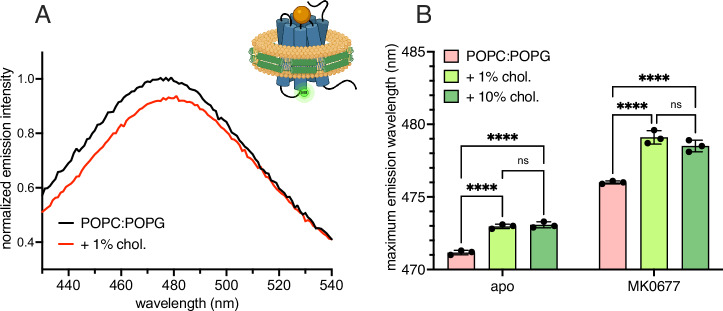
Effect of lipids on the GHSR conformational equilibria. **A** normalized MB emission spectra of the GHSR in the absence or presence of 1% cholesterol in the nanodiscs and in the presence of 10 µM MK0677. **B** Maximum emission wavelength of MB in the absence or presence of 10 µM MK0677 and in the presence of 1% or 10% cholesterol in the nanodiscs (see Fig. S4 for the corresponding emission spectra). The spectra shown in (**A**) are representative of one of three independent experiments. Data in **B** are mean ± SD of three replicates per group. ns not significant, and ****p ≤ 0.0001, by one-way ANOVA test with Bonferroni post-test. Inset created in BioRender. BANERES, J. (2026) https://BioRender.com/dldcve3.

We first used monobromobimane (MB) fluorescence to analyze the effect of cholesterol on the GHSR conformation. To this end, the ghrelin receptor was assembled into POPC:POPG nanodiscs containing varying amounts of cholesterol (1 or 10 mol%, noted 1% and 10% in the subsequent sections). As stated in the Materials & Methods section, cholesterol was added to the other lipids prior to evaporation and reconstitution, as previous reports showed that this yields nanodiscs with a cholesterol content consistent with the target concentration^18^. A 10% value was used as the upper limit in all subsequent experiments, as in our hands homogeneous preparations could not be obtained for cholesterol amounts above this value. Under all conditions, the receptor was functional, as assessed in a GTP turnover assay with the purified heterotrimeric Gα_q_β_1_γ_2_ (Fig. S1). To be noted, a slight but significant increase in GTP binding to Gq was observed in the presence of cholesterol in the nanodiscs, consistent with our previous report with Gi^16^. For the MB fluorescence experiments, we used a minimal cysteine GHSR mutant where all intrinsic reactive cysteines, i.e. C146^3.55^ and C304^7.34^ (superscript numbers refer to the Ballesteros-Weinstein numbering^19^), were replaced with serines^20^. We previously showed that none of the remaining cysteines are accessible to fluorophores^20^. We then introduced a unique reactive cysteine at position 255^6.27^ for labeling with MB, as a reporter of TM6 movement associated with receptor activation^21^. Of note, none of these mutations affected the pharmacological properties of the GHSR, at least as far as ligand binding is considered (Fig. S2). Using this assay, we previously showed that adding PIP2 in the nanodiscs affected the conformational equilibrium of the GHSR, favoring the active/active-like states^21^ (see also Fig. S3). As shown in Figs. 1A, S4, the addition of cholesterol to the nanodiscs affected the MB emission spectrum in the same way. Specifically, a decrease in the emission intensity and a red shift of the maximum emission wavelength were observed in the presence of cholesterol, suggesting that this lipid also shifted the conformational equilibrium of the GHSR away from the inactive state. This occurred with both the apo- and agonist-loaded receptor, suggesting that cholesterol affected the conformational equilibrium of the receptor irrespective of the presence of the ligand. However, the extent of the change in the MB emission properties was lower than with PIP2 (Fig. S3), suggesting cholesterol was less efficient at shifting the GHSR conformational equilibrium. Finally, the effect of cholesterol on the MB emission spectra was similar at low and high mol% (Figs.1B, S4), indicative of a saturable effect. To be noted, these effects are likely due to PIP2 or cholesterol rather than to the negatively charged POPG lipid, as the same trend was observed with nanodiscs composed of POPC only instead of the POPC:POPG mixture (Fig. S5).

### Effect of PIP2 and cholesterol in the presence of signaling proteins

**Fig. 2:**
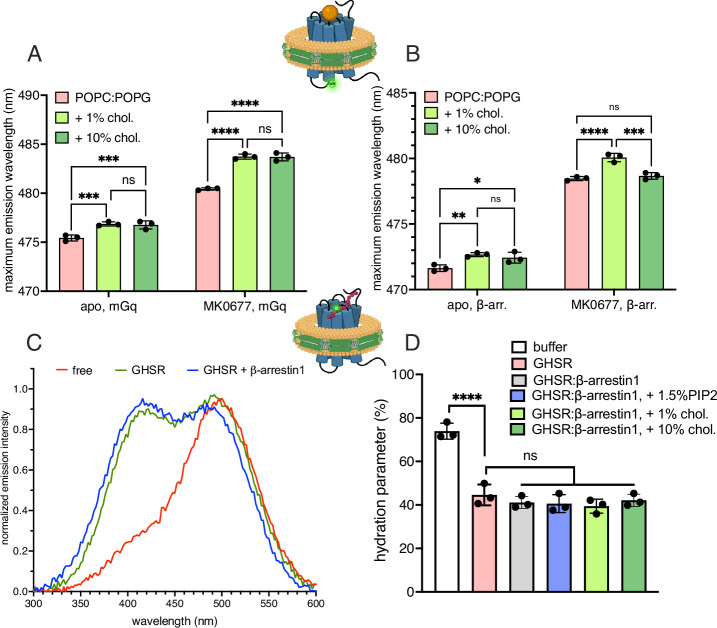
Effect of signaling proteins on the GHSR conformation. **A** Maximum emission wavelength of MB in the presence of mGq, in the absence or in the presence of 10 µM MK0677, and in the presence of either 1% or 10% cholesterol in the nanodiscs. **B** Same as in (**A**) but in the presence of ΔCter β-arrestin1 instead of mGq. The emission spectra for (**A**) and (**B**) are given in Figure S6. **C** Normalized emission spectra of 7-H4MC-labeled ghrelin(1-10), in the absence or presence of GHSR-containing POPC:POPG nanodiscs and ΔCter β-arrestin1. **D** Hydration parameter inferred from the 7-H4MC emission spectra. Spectra in **C** are normalized and representative of one of three independent experiments. Data in (**A**, **B**, **D**) are mean ± SD of three replicates per group. ns: not significant, *p ≤ 0.05, **p ≤ 0.01, ***p ≤ 0.001 and ****p ≤ 0.0001, by one-way ANOVA test with Bonferroni post-test. Insets created in BioRender. BANERES, J. (2026) https://BioRender.com/dldcve3.

We then investigated the impact of G proteins and β-arrestin1 on the conformation of the GHSR, and whether PIP2 or cholesterol modulated this impact. We used here mini-Gs/q71 as a Gq surrogate, since this engineered variant of Gαq is better suited for biophysical studies^22^ and stabilizes the receptor active state as the full heterotrimer does, as evidenced in its cryo-EM structure of the complex with the GHSR^15^. Mini-Gs/q71 will be referred to as mGq throughout the text. Addition of mGq to the receptor in POPC:POPG nanodiscs further altered the MB emission profile both in the absence of ligand and in the presence of MK0677 (Fig. S3), consistent with our previous data with the Gα_q_β_1_γ_2_ trimer^21^. In the presence of either 1.5% PIP2 (Fig. S3) or 1% cholesterol (Figs.2A, S6A), a further red shift in MB emission was observed compared to POPC:POPG-only nanodiscs for both apo- and agonist-loaded states, suggesting that mGq and PIP2 or cholesterol cooperate for stabilizing the active conformation of the GHSR. Of note, the increase in the λ_max_ of MB emission was of smaller amplitude for cholesterol than for PIP2 (Fig. S3). Finally, no further effect was observed when the nanodiscs contained 10% cholesterol compared to 1% (Figs.2A, S6A), indicating that, as in the case of the isolated receptor, the effect of cholesterol was saturable.

We then analyzed the effect of β-arrestin1 on the GHSR conformation. To this end, the isolated receptor in nanodiscs was first phosphorylated in vitro with recombinant GRK5 in the presence of the full agonist MK0677. We previously showed that the GHSR phosphorylated with this recombinant version of GRK5^23^ recruited β-arrestin1 in a similar manner than a receptor where a synthetic C-terminal peptide with all known GHSR phosphosites in their phosphorylated state^24^ had been enzymatically fused to the GHSR core^25^. In addition, we used a modified version of β-arrestin1 that is constitutively active due to the truncation of its C-terminal region (ΔCter β-arrestin1)^26^. The MB emission profiles in the presence of ΔCter β-arrestin1 were significantly different from those observed with mGq. First, in the absence of MK0677, the changes in MB emission were closely related to those observed in the absence of effector (Figs.2B; S6B). This suggests a limited basal recruitment of β-arrestin or, alternatively, that the interaction in the absence of agonist has limited effect on the conformational features of the GHSR. In contrast, ΔCter β-arrestin1 promoted a change in the MB emission properties in the MK0677-loaded state (Figs.2B; S6B). This change was further enhanced upon addition to the nanodiscs of either 1.5% PIP2 (Figs.S3) or 1% cholesterol (Figs.2B; S6B). However, in contrast to mGq, the effect of β-arrestin1 in the agonist-loaded state was highly dependent on the amount of cholesterol in the nanodiscs. Indeed, whereas a significant effect was observed in the presence of 1% cholesterol, the MB emission profile in the presence of 10% cholesterol was essentially indistinguishable from that observed with POPC:POPG nanodiscs (Figs.2B; S6B). This suggests that ΔC β-arrestin1 was inefficient at further shifting the inactive-to-active conformational equilibrium of the GHSR at this higher cholesterol-to-lipid molar ratio. Of note also, the effect ΔC β-arrestin1 on the GHSR conformational features was less pronounced than that induced by mGq regardless of the lipid composition (Fig. S7).

We recently used the fluorescent unnatural amino acid (UAA) L-(7-hydroxycoumarin-4-yl)-ethylglycine (7-H4MC) to monitor the accessibility of ghrelin to the solvent when located in its binding site^27^. We used this strategy to investigate whether binding to β-arrestin1 affected the GHSR conformation in a similar way. To this end, we analyzed the emission profiles of 7-H4MC at position 4 of ghrelin using an excitation wavelength of 320 nm to excite the neutral form of the fluorophore and determined a hydration parameter H from the resulting emission spectra^28^. In contrast to what we reported for the Gq peptidomimetic, the emission spectra and associated hydration parameter were similar in the absence or presence of β-arrestin1, whatever the lipid composition of the nanodisc was, indicative of a similar solvent accessibility in all conditions (Fig.2C, D). This suggests that the arrangement of the extracellular ligand binding pocket of the GHSR was not significantly affected by the coupling to β-arrestin1, in contrast to what occurs with the G protein surrogate^27^.

### Effect of cholesterol on G protein recruitment

**Fig. 3:**
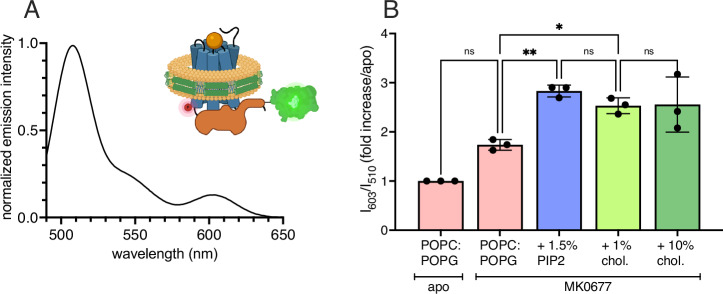
Effect of lipids on mini-G protein recruitment to the GHSR. **A** Normalized emission spectrum of the AlexaFluor 568-labeled GHSR in POPC:POPG nanodiscs in the presence of GFP-mGq protein (receptor-to-mGq molar ratio 1:2.5). **B** Acceptor-donor emission intensity ratio changes in the absence of ligand or in the presence of 10 µM MK0677, and in the presence of either 1.5% PIP2, 1% or 10% cholesterol in the nanodisc. Changes are reported to the ratio obtained for the apo-receptor in POPC:POPG nanodiscs (see Fig. S8 for the corresponding spectra). The spectrum shown in (**A**) is normalized to the maximum emission intensity and representative of one of three independent experiments. Data in **B** are mean ± SD of three replicates per group. ns: not significant, *p ≤ 0.05 and **p ≤ 0.01, by one-way ANOVA test with Bonferroni post-test. Inset created in BioRender. BANERES, J. (2026) https://BioRender.com/dldcve3.

We previously demonstrated that including PIP2 into the nanodiscs increased GHSR-catalyzed Gq activation, based on a GTP turnover assay^21^. To assess whether this resulted from an increase in the population of the receptor:G protein complex or from an effect of PIP2 on the G protein guanine nucleotide exchange factor activity, we monitored the formation of the GHSR:mGq assembly using a FRET-based assay. In this case, mGq was fused to GFP at its N-terminus, as this modification was reported not to affect its coupling to isolated receptors^22^. In parallel, the ghrelin receptor was labeled with Alexa Fluor 568 on a unique reactive cysteine at position 71^1.60^. As in the case of the cysteine at position 255^6.27^, C71^1.60^ was introduced in the minimal cysteine mutant of the GHSR, and this modification did not affect the ligand binding properties of the resulting receptor (Fig. S2)^29^. Under such conditions, a FRET signal was observed with the apo receptor (Figs.3; S8), indicative of an interaction even in the absence of agonist. This is consistent with the GHSR:G protein pre-association we reported to occur with the Gα_q_β_1_γ_2_ heterotrimer^29^. An increase in the FRET ratio was then observed upon MK0677 binding to the receptor, indicative of a further recruitment of mGq to the receptor (Figs.3B; S8). In the presence of 1.5% PIP2 in the nanodiscs, a further increase in the FRET ratio was observed (Figs.3B; S8), possibly reflecting an increase in mGq protein recruitment. Of note, this increase mirrored the larger receptor-catalyzed G protein activation we previously reported to occur under similar conditions^21^. A similar effect was observed with nanodiscs containing different amounts of cholesterol (Fig.3B; S8). This suggests that cholesterol also promoted mGq recruitment to the GHSR, which might be related to its impact on GTP turnover and GHSR conformation.

### Effect of cholesterol on β-arrestin1 recruitment

**Fig. 4:**
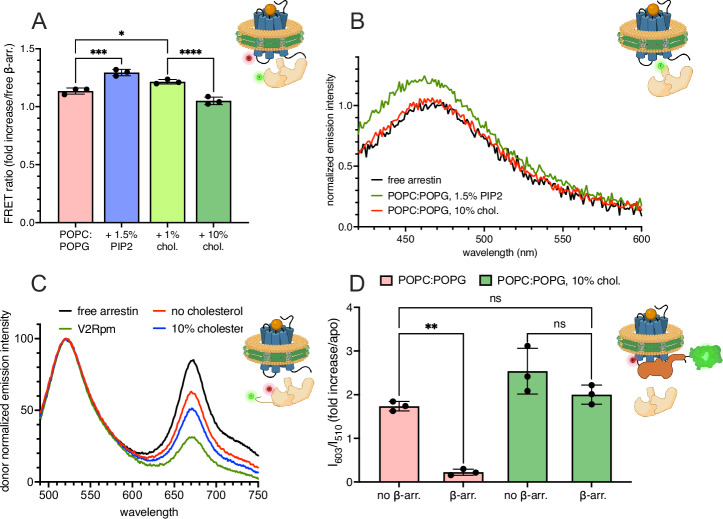
Effect of cholesterol on β-arrestin1 coupling to the GHSR. **A** Changes in the FRET ratio with the GHSR labeled with AlexaFluor 350 and β-arrestin1 labeled with AlexaFluor 488 in the presence of 10 µM MK0677 and in the absence or presence of 1.5% PIP2 or 1% or 10% cholesterol in the nanodiscs. **B** MB emission spectra of ΔCter β-arrestin1 labeled with bimane at position 68 in the absence of nanodiscs (free arrestin) or in the presence of the GHSR, with 10 µM MK0677 and 1% PIP2 or 10% cholesterol. **C** Emission spectra of wild-type β-arrestin1 labeled with AlexaFluor 488 and AlexaFluor 647 in the free state, in the presence of 50 µM V2R C-tail phosphomimetic peptide, or in the presence of nanodiscs containing the GRK5-phosphorylated GHSR, in the absence or in the presence of 10% cholesterol. Spectra are normalized to the donor intensity within each experiment. **D** GHSR-mGq acceptor-donor emission intensity ratio in the presence of 10 µM MK0677, in the absence or presence of 10% cholesterol, and in the absence or presence of a 50-fold excess in β-arrestin1. Data in the absence of β-arrestin1 is from Fig. 3. Values in (**A**, **D**) are mean ± SD of three replicates per group ns: not significant, *p ≤ 0.05, ***p ≤ 0.001 and ****p ≤ 0.0001, by one-way ANOVA test with Bonferroni post-test. In (**B**, **C**), the spectra are normalized and representative of one of three independent experiments. Inset created in BioRender. BANERES, J. (2026) https://BioRender.com/dldcve3.

We recently showed that PIP2 promoted β-arrestin1 recruitment to the agonist-activated GHSR^25^. We further analyzed here whether cholesterol had a similar effect. To this end, we devised an assay based on the measurement of the FRET signal between the GHSR labeled with the fluorescent donor Alexa Fluor 350 on C71^1.60^ and the β-arrestin1 labeled on a unique reactive cysteine at position 167, i.e. in its N-lobe, with the acceptor Alexa Fluor 488. This FRET signal was recently shown to report on receptor:effector interaction^25^. To be noted, this position is closely related to the F2, F4-5 labeling positions that have been recently described to report on conformational changes in the phosphorylation-sensing N-domain of β-arrestins^30^. To avoid any bias that could be associated with β-arrestin truncation, wild-type β-arrestin1 was used here instead of its ΔCter version. As shown in Fig. 4A, when labeled β-arrestin1 was added to the agonist-loaded, GRK5-phosphorylated GHSR, a FRET signal was observed, indicating an interaction between both partners. This was not observed in the presence of JMV2959, which is a partial agonist for Gq activation but an antagonist for β-arrestin recruitment to the GHSR (Fig. S9A)^31^. Consistent with our recent data in bimane-based experiments^25^, an increase in the FRET ratio was observed when the nanodiscs contained 1.5% PIP2 (Fig.4A). In the presence of 1% cholesterol, a slight increase in the FRET ratio was also observed, although this increase was of lower amplitude compared to that observed with PIP2 (Fig.4A). Despite a different geometric arrangement cannot be excluded at this stage, this increase could be due to an increase in the population of the complex in the presence of 1% cholesterol compared to POPC:POPG only nanodiscs. Indeed, we recently showed that the LRET signal for the 71-167 pair was not sensitive to the changes in the arrangement of the GHSR:β-arrestin1 complex^25^. However, when the nanodiscs contained 10% cholesterol, a significantly lower FRET ratio was measured. The same trend was observed when β-arrestin1 was labeled on its C-lobe (C191) instead of the N-lobe (Fig. S9B). Of note, the reduced FRET signal under such conditions was likely associated to a rearrangement of the GHSR:β-arrestin1 assembly rather than to a dissociation of the complex. Indeed, an analytical size-exclusion chromatography analysis indicated the persistence of a complex even at high cholesterol content in the nanodiscs (Fig. S10).

### Effect of cholesterol on the arrangement of the GHSR:β-arrestin1 complex

To assess whether the effect observed on the FRET signal at 10% cholesterol was due to a rearrangement of the GHSR:β-arrestin1 complex, we further explored the organization of this assembly in the presence of different cholesterol amounts in the nanodiscs. β-arrestins have been proposed to interact with activated, phosphorylated GPCRs in two different manners^32^. The first one involves an interaction of the N-lobe only with the phosphorylated C-terminus of the receptor (tail engagement), while the second involves additional contacts with the receptor TM domains (core engagement). We used here two different assays to monitor which kind of complex might occur with the isolated ghrelin receptor. To monitor core engagement, we labeled β-arrestin1 with the environmentally sensitive fluorescent probe MB on a unique reactive cysteine at position 68 in the finger loop^33^. The latter is a key component of the core interaction with the receptor^34^. In all cases, 1.5% PIP2 was added to the nanodiscs, as this has been shown to favor β-arrestin recruitment^35^. As shown in Fig. 4B, a significant change in the MB emission intensity was observed for nanodiscs containing the GHSR and no cholesterol. The changes affected essentially the emission intensity, with a slight blue-shift in the maximum emission wavelength, suggesting a possible alteration of the physicochemical properties of the probe environment that would result from its insertion of the finger loop into the receptor core. In contrast, essentially no change in the emission properties of the fluorescent reporter was observed when the nanodiscs included 10% cholesterol (Fig.4B). This is indicative of a significantly reduced insertion of the β-arrestin1 finger loop into the GHSR TM core in the presence of high cholesterol amounts in the nanodiscs.

We then visualized the tail engagement using the fluorescent assay recently described^35^. This assay relied on the introduction of two fluorophores (AlexaFluor 488 and 647) through unique reactive cysteines at positions 12 and 387 of purified β-arrestin1. The resulting FRET signal reports on the release of the β-arrestin1 C-terminus from its N-domain that occurs upon binding of the phosphorylated GPCR C-terminus. We first used as a positive control a phosphomimetic of the C-terminus of the vasopressin V2 receptor that was demonstrated to interact with recombinant β-arrestin1^36^. In this case, a decrease in the FRET signal was observed, indicative of a release of the β-arrestin1 C-tail (Fig.4C). A significant decrease in the FRET signal was also observed with the GRK5-phosphorylated GHSR, both in the absence and in the presence of high mol% cholesterol in the lipid nanodiscs (Fig.4C). To be noted, no change in the FRET signal was observed with the unphosphorylated receptor, whether the nanodiscs included cholesterol or not (Figure S11). Altogether, these data point to a model where β-arrestin1 would interact with the phosphorylated receptor both at the core and C-tail in the absence of cholesterol, while it would adopt the tail-only engagement configuration in the presence of 10% cholesterol. Accordingly, adding an excess of the V2R-phosphomimetic peptide led to a significant dissociation of the complex only in the presence of 10% cholesterol in the nanodiscs (Fig. S12), which was not the case in the absence of cholesterol where other contact might contribute to the stability of the complex. Of note, the latter observation was consistent with recent data with the β1-adrenergic receptor where a complex with β-arrestin was observed in vitro in the presence of an exogeneous phosphorylated V2R C-terminal peptide^37^.

### Effect of cholesterol on the GHSR:G protein:β-arrestin1 interplay

GPCR desensitization has been shown to involve the core-engaged conformation^38^. To assess whether this could be the case here, we compared the GHSR:mGq FRET signal in the absence or presence of β-arrestin1 and in the absence or presence of 10% cholesterol, i.e., under conditions where either the core- or the tail-conformations would prevail. As shown in Fig. 4D, in the absence of cholesterol in the nanodiscs, adding an excess of β-arrestin1 to the GHSR:mGq complex was associated to a significant decrease in the FRET signal, suggesting a dissociation of the GHSR:mGq assembly under such conditions. In contrast, a much smaller decrease in the FRET signal was observed in the presence of 10% cholesterol in the nanodiscs (Fig.4D), i.e., under conditions where β-arrestin1 essentially interacted with the GHSR C-tail. Taken together, these data indicate that, in our reconstituted system, a core interaction between the GHSR and β-arrestin1 was essential for dissociating the G protein surrogate from the receptor. A super-complex such as the one previously described for other GPCRs^39^ could be responsible for what was obtained here at high cholesterol content, where both mGq and β-arrestin1 would interact with the GHSR.

### Effect of cholesterol on the interaction of β-arrestin1 with the lipid bilayer

**Fig. 5:**
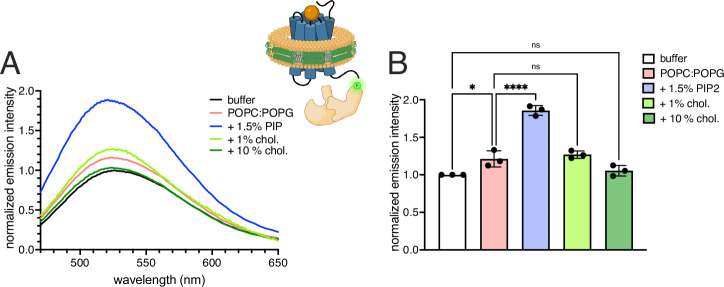
Effect of cholesterol on β-arrestin1 interaction with the bilayer. **A** DNS emission spectra of ΔCter β-arrestin1 labeled on C341 free in solution (buffer) or in the presence of GHSR-nanodiscs containing either 1.5% PIP2, 1% cholesterol, or 10% cholesterol. **B** DNS normalized maximum emission intensity of ΔCter β-arrestin1 labeled on C341 free in solution or in nanodiscs of different lipid composition. In **B** values are mean ± SD of three replicates per group, with ns not significant, *p ≤ 0.05, and ****p ≤ 0.0001, by one-way ANOVA test with Bonferroni post-test. The emission spectra in (**A**) were normalized to the maximal emission intensity observed in the absence of nanodiscs and are representative of one of three independent experiments. Inset created in BioRender. BANERES, J. (2026) https://BioRender.com/dldcve3.

As the membrane plays a central role in the coupling of β-arrestin1 to GPCRs^25,35,40^, we then analyzed whether the decrease in the interaction of β-arrestin1 with the GHSR TM core could be associated to a decrease in its binding to the lipid bilayer of the nanodiscs at the highest cholesterol % used. To this end, we introduced a unique reactive cysteine in the C-edge loop of β-arrestin1 (S341) and labeled it with N-[2-(Dansylamino)ethyl]maleimide (DNS). DNS is an environmentally-sensitive fluorophore whose emission properties vary upon insertion into a hydrophobic environment such as the lipid bilayer^41^. This labeling position was selected based on previous reports that the C-edge loop of β-arrestins is a region directly interacting with the lipid bilayer^42^. A change in the emission properties of DNS attached to C341 of β-arrestin1 was observed in POPC:POPG nanodiscs, indicating loop insertion into the bilayer (Fig.5A, B). A further increase was observed when 1.5% PIP2 was added to the nanodiscs, indicative of a larger membrane-interacting population (Fig.5A,B). Including cholesterol in the nanodiscs at 1% was also associated to an increase in the emission intensity of DNS, although to a lower extent than in the presence of PIP2 (Fig.5B). This might be the consequence of an increased binding to the receptor, as no significant change was observed with receptor free nanodiscs (Fig. S13A). In contrast, no major change in the DNS emission profile was observed at the highest lipid-to-cholesterol ratio (Fig.5B), even in the presence of 1.5% PIP2 (Fig. S13B), suggesting a reduced insertion of the C-edge loop into the nanodisc lipid bilayer. This could be due to a change in the lipid order of the nanodisc bilayer in the presence of high amounts of cholesterol, as suggested by Laurdan fluorescence emission experiments (Fig. S14).

## Discussion

**Fig. 6:**
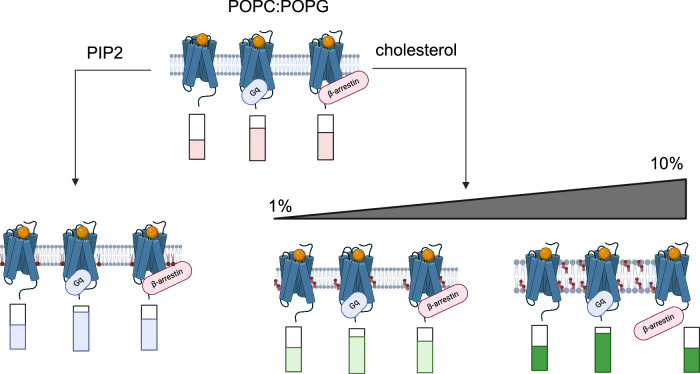
Schematic representation of the impact of PIP2 and cholesterol on the GHSR conformation and interaction with its effectors. The bars under each species represent the relative proportion of inactive (white) and active (colored) states. Created in BioRender. BANERES, J. (2026) https://BioRender.com/ff9rssg.

There is increasing evidence that the membrane environment influences the structural and functional properties of GPCRs both through its bulk properties and through specific protein-lipid interactions^4^. Using fluorescence spectroscopy and nanodiscs as a membrane model, we provide here evidence that cholesterol shifts the conformational equilibrium of the ghrelin receptor toward its active/active-like states (Fig.6). Moreover, this particular lipid affects the coupling of the ghrelin receptor isolated in lipid nanodiscs to its signaling partners in different ways, depending on the effector considered (Fig.6). On the one hand, it stabilizes the complex with the G protein surrogate mini-Gq, whatever its concentration in the nanodiscs (Fig.6). On the other hand, possibly by affecting the interplay of β-arrestin1 with the lipid bilayer, it is associated with a shift in the organization of the complex that this effector forms with the GHSR, from a core- to a tail-only engagement (Fig.6).

Among all the lipids found in the plasma membrane, cholesterol is one of the most abundant and a potent modulator of GPCR function^4^. Consistently, our data here show that this particular lipid affects the conformational equilibrium of the GHSR in the absence of any effector, favoring its active/active-like states. This suggests that cholesterol might lower the energy barrier between the inactive and active/active like GHSR states, although it appears less efficient at doing so than another key component of the membrane, PIP2.

The effect of cholesterol on the GHSR conformational equilibrium appears to be primarily due to a direct interaction with the receptor, as reported for the A2AAR^18^. Indeed, as in the case of this receptor, the changes in MB emission occurred at low mol% values. This is consistent with the observation that, in cryo-EM structures, cholesterol analogues were systematically found closely associated with the transmembrane regions of the ghrelin receptor when bound to agonists and G proteins^15,16^. This does not exclude an additional contribution of the cholesterol effect on the bulk properties of the bilayer at higher mol%, however. Indeed, we previously showed that the conformational equilibrium of the GHSR depended on the membrane thickness^21^, and cholesterol has been found to directly affect this thickness^43^. Therefore, the sensitivity of the GHSR conformation to cholesterol might as well result from a combination of its direct interaction with the GHSR and of its effect on the bulk properties of the bilayer.

Our MB emission data indicated that the agonist alone stabilized the active state of the receptor, but that both the G protein surrogate and β-arrestin1 could further shift the conformational equilibrium towards the active conformation. Of importance, the effect of β-arrestin1 on MB emission properties was of significantly lower extent than that observed for the mini-G protein. A similar observation was reported for the β2-adrenergic receptor^44^. Although we cannot exclude that this effector stabilizes a different conformational state with distinct MB emission properties, this nevertheless suggests that the effect of β-arrestins on the GHSR conformation is less pronounced than that of the G protein, in agreement with recent observations with the µ-opioid receptor^45^. In addition, whereas binding of G protein surrogates allosterically affected the geometric features of the distant GHSR ligand binding pocket at the extracellular part of the receptor^27^, β-arrestin1 did not, at least under the conditions of the assay used here, in accordance with the differences in the ligand binding pocket and ligand-receptor contacts between the β1-adrenergic receptor coupled to a Gs protein mimetic or β-arrestin^46^. This again suggests that despite binding to a related pocket in the receptor TM core, the G protein and β-arrestin1 differ in their allosteric effect on the GHSR structural features.

In our isolated system, cholesterol appeared to have a different effect on the formation of signaling complexes depending on whether the G protein or β-arrestin1 was involved. Cholesterol favored the GHSR:mini-G protein interaction regardless of its content in the nanodiscs. In contrast, the effect of cholesterol on the interaction with β-arrestin1 depended on its amount in the nanodiscs. Indeed, we observed a shift from a core organization at low cholesterol levels, where β-arrestin1 interacted with both the TM core of the receptor and its phosphorylated C-terminus, to a C-tail organization at higher cholesterol levels, where β-arrestin1 interacted only with the C-terminus of the GHSR. This is unlikely to be due to a different effect of cholesterol on the GHSR conformation, since the emission of MB bound to the receptor was similar regardless of the mol% of cholesterol. Rather, our data indicate a concomitant dissociation of the C-edge loop of β-arrestin1 from the lipid bilayer of the nanodiscs as the tail-bound complex became the major one in solution. Increasing cholesterol increases acyl chain order and decreases membrane water permeability of lipid bilayers^43^. One possibility would thus be that such an increase in lipid order in the nanodiscs limits membrane insertion of the β-arrestin1 C-edge loop. A reduced interaction of the C-edge loop with the lipid bilayer could then affect the interaction of the finger loop with the receptor core, possibly by decreasing the global affinity of β-arrestin for the membrane-embedded receptor, leading to the formation of the tail-only engaged complex. This would be consistent with previous observations where the interaction of β-arrestin1 with the membrane *via* its C-edge loops favored high-affinity core interactions^47^.

The altered interaction of the GHSR with β-arrestin1 in the presence of high cholesterol amounts is puzzling in the context of receptor internalization. Indeed, this process, which in many cases involves interaction with β-arrestins, typically occurs into cholesterol-rich membrane domains^48^. A possibility would be that the tail-only engagement observed at high cholesterol content might as well be sufficient to drive internalization of the GHSR. Indeed, previous reports indicated that the engagement of only the phosphorylated tail of GPCRs was sufficient to mediate receptor internalization and ERK/MAPK activation^34,38,49,50^. More recently, data on the glucagon receptor GCGR also showed that the tail-only conformation governed β-arrestin recruitment and endocytosis of this receptor^51^. In all these reports, only desensitization of G protein signaling appeared to require the additional engagement of the receptor core^38^, in agreement with what we observed here in the competition assay between the mini-G protein and β-arrestin1 for GHSR binding. Hence, this would favor a model where a functional segregation between the tail- and the core-engaged complexes would be at play, with a contribution of the membrane. Regardless of the details, our work reinforces the paradigm in which the bilayer properties, the receptor conformational landscape and the functional fate are tightly coupled, suggesting that alterations in membrane composition can directly influence receptor behavior and signaling outcomes.

## Methods

### Materials

All chemicals were purchased from Sigma-Aldrich (Darmstadt, Germany), except for β-DDM, which was obtained from Anatrace (Maumee, OH, USA). All lipids were from Avanti Polar (Alabaster, AL).

### Protein expression and purification

*GHSR—*The human ghrelin receptor GHSR fused to the α_5_ integrin was expressed in *E. Coli BL21* inclusion bodies^33^. Rosetta(DE3) cells were transformed with the pET21a-GHSR plasmid and grown in Terrific Broth (TB) at 37 °C supplemented with 100 µg/mL carbenicillin. Protein expression was induced with 1 mM IPTG. After 4 h induction, cells were harvested (5400 x *g*, 30 min.) and purification of the receptor under denaturing condition was achieved as previously described^33^. Briefly, after resuspension in lysis buffer (50 mM Tris, 2 M urea, 0.5 mM PMSF and EDTA-free protease inhibitor cocktail), cell lysis by sonication, and centrifugation (27,000 x *g*, 40 min.), inclusions bodies were solubilized in a buffer 100 mM NaH_2_PO_4_, 10 mM Tris-HCl pH 8.0, 6 M urea, 10% glycerol, 1% SDS, 1 mM TCEP, 10 mM imidazole and loaded on a Ni-NTA superflow resin (Qiagen). IMAC purification was then carried out with two washing steps at 10 mM and 20 mM imidazole, and elution at 200 mM imidazole. The α5 integrin fusion partner was removed by thrombin cleavage (0.5% (w/w) thrombin) under non-denaturing conditions, i.e. in a 50 mM Tris-HCl pH 8, 150 mM NaCl, 2.5 mM CaCl_2_ buffer. Thrombin activity was stopped by adding 6 M urea, 1 mM TCEP, 10% glycerol, 0.8% SDS, and 10 mM imidazole (final concentrations). The GHSR was recovered in the flow-through fractions after loading a Ni-NTA Superflow column and dialyzed against a 50 mM Tris-HCl pH 8.0, 1.0% SDS, 1 mM TCEP buffer.

*MSP1E3D1(-)—*The MSP1E3D1 protein was expressed and purified as described^52^. BL21(DE3) transformed with the pET28-MSP1E3D1 encoding plasmid was grown in TB medium supplemented with 100 µg/mL kanamycin at 37 °C and protein expression was induced by adding 1 mM IPTG. After 4 h induction at 26 °C, cells were harvested (5400 x *g*, 30 min.) and resuspended in a 20 mM sodium phosphate buffer at pH 7.4, containing an EDTA-free protease inhibitor cocktail. Cells lysis was achieved by sonication in presence of 1% Triton X-100 and the lysate was clarified by centrifugation (27,000 x *g*, 40 min.). The supernatant was loaded onto a Ni-NTA superflow resin and IMAC purification was achieved by three successive washes with buffers 40 mM Tris-HCl pH 8.0, 300 mM NaCl, 1% Triton X-100; 40 mM Tris-HCl pH 8.0, 300 mM NaCl, 50 mM Na-cholate, 20 mM imidazole; 40 mM Tris-HCl pH 8.0, 300 mM NaCl, 50 mM imidazole, and elution with 40 mM Tris/HCl pH 8.0, 300 mM NaCl, 400 mM imidazole. Elution fractions were dialyzed against 50 mM Tris-HCl pH 8.0, 0.5 mM EDTA buffer before overnight digestion with TEV protease (1:100 ratio (w/w)) in the presence of 1 mM DTT. MSP1E3D1(-) was finally recovered by reverse IMAC purification and extensively dialyzed against storage buffer (20 mM Tris-HCl, pH 7.5, 100 mM NaCl, 0.5 mM EDTA, 0.03% NaN_3_).

*GRK5*—GRK5 was expressed and purified as reported^23^. The plasmid encoding GRK5 was a generous gift of Dr. J.J.G. Tesmer. *E. coli* Rosetta(DE3) *cells* transformed with the pEL17 plasmid encoding for GRK5 were grown in TB medium at 37 °C supplemented with 100 µg/mL carbenicillin and then cooled to 18 °C. Protein expression was induced overnight at 18 °C by adding 0.2 mM IPTG. After centrifugation (5400 x *g*, 30 min.), cell pellet was resuspended in lysis buffer (20 mM HEPES pH 8.0, 200 mM NaCl, 40 mM imidazole, 5 mM MgCl_2_, 5 mM CaCl_2_, supplemented with an EDTA-free protease inhibitor cocktail), sonicated and Triton X-100 was added (0,02%, v/v). The lysate was clarified by centrifugation (27,000 x *g*, 40 min.) and the supernatant was loaded onto a Ni-NTA superflow resin (Qiagen), washed with the lysis buffer and eluted with the lysis buffer containing 200 mM imidazole. The eluted protein was dialyzed into a 20 mM HEPES pH 8.0, 100 mM NaCl, 1 mM DTT buffer and loaded on an anionic followed by a cationic exchange column. GRK5 protein was then eluted using a NaCl gradient in previous buffer from 100 to 500 mM, dialyzed against a 20 mM Na-HEPES pH 8.0, 200 mM NaCl, 1 mM DTT buffer and loaded on a on Superdex 200 increase 10/300 GL column (Cytiva) with the same buffer as the eluent.

*β-arrestin1—*The full-length cysteine-free β-arrestin1 mutant and its pre-activated ΔCter version (β-arrestin (1-382)) with reactive cysteines at positions 68, 167 or 341, or β-arrestin1 with reactive cysteines at positions 12 and 387 were produced as recently described^36^. The initial construct was human β-arrestin1 in which all cysteine residues were removed by site-directed mutagenesis (C59V, C125S, C140L, C150V, C242V, C251V, C269S). ΔCter β-arrestin1 was prepared from this construct by truncating the protein at residue 382. Both constructs were then modified by introducing a cysteine at the above-mentioned positions by site-directed mutagenesis, and adding an N-terminal hexahistidine tag followed by a 3 C protease site. The sequence was codon-optimized for expression in *E. coli* and cloned into a pET-15b vector (Genecust). *E. coli* NiCo21(DE3) cells (NEB) were transformed with the resulting expression vector. Cultures were initially grown in 2YT medium supplemented with 100 µg/mL carbenicillin at 37 °C. When the culture reached mid-log phase, the temperature of the culture was decreased to 16 °C and protein expression induced with 25 μM IPTG for 20 hours. Cells were harvested, resuspended in 50 mM Tris-HCl pH 8, 300 mM NaCl, 15% glycerol, 1 mM TCEP supplemented with an EDTA-free protease inhibitor cocktail (Roche), lysed by sonication, and centrifuged at 27,000 x *g* for 45 min. The supernatant was applied to a HisTrap 5 mL column (Cytiva) and washed with a 50 mM Tris-HCl pH 8, 300 mM NaCl, 15% glycerol, 1 mM TCEP buffer containing 20 mM and then 40 mM imidazole. The protein was eluted with the same buffer containing 200 mM imidazole, dialyzed into a 25 mM Na-HEPES, 200 mM NaCl, 1 mM TCEP, 10% glycerol, pH 7.5 buffer, digested with 3 C protease (16 h at 20 °C) and subjected to reverse-nickel purification. The resulting protein was then diluted with a buffer lacking NaCl to a final NaCl concentration of 40 mM, loaded on a HiSure Q 5 mL column (Cytiva), and eluted with a linear 40 to 400 mM NaCl gradient in 25 mM Na-HEPES, 1 mM TCEP, 10% glycerol, pH 7.5. The recovered protein was finally subjected to size-exclusion chromatography using a Superdex 200 increase 10/300 GL column (Cytiva) in 20 mM HEPES pH7.5, 200 mM NaCl, 10% glycerol, 1 mM TCEP.

*Mini-Gs/q71—*The mini-Gs/q71 protein (named here mGq) was expressed and purified as described^53^. BL21(DE3) cells transformed with a pET28 plasmid encoding mGq (Genecust) were grown in TB medium supplemented with 0.2% glucose and 100 µg/mL kanamycin at 30 °C, and protein expression was induced overnight at 25 °C by adding 50 µM IPTG. Cells were then harvested (5400 x *g*, 30 min.) and resuspended in a 40 mM Na-HEPES pH 7.5 buffer containing 100 mM NaCl, 10 mM imidazole, 10% Glycerol, 5 mM MgCl_2_, 0.05 mM GDP, 1 mM PMSF supplemented with an EDTA-free protease inhibitor cocktail. Cells lysis was achieved by sonication and the lysate clarified by centrifugation (27,000 x *g*, 40 min.). The supernatant was loaded onto a 5 mL HisTrap column (Cytiva). The column was washed with a buffer 20 mM Na-HEPES pH 7.5, 500 mM NaCl, 40 mM imidazole, 10% Glycerol, 1 mM MgCl_2_, 0.05 mM GDP, 1 mM PMSF and mGq protein was eluted with a 20 mM Na-HEPES pH 7.5, 100 mM NaCl, 500 mM imidazole, 10% Glycerol, 1 mM MgCl_2_, 0.05 mM GDP, 1 mM PMSF buffer. The Histag was removed by TEV protease digestion (1:20 ratio, w/w) in the presence of 1 mM DTT during an overnight dialysis against a 20 mM Na-HEPES pH 7.5, 100 mM NaCl, 10% Glycerol, 1 mM MgCl_2_, 0.05 mM GDP buffer. mGq was then recovered by reverse IMAC purification, extensively dialyzed against 10 mM Na-HEPES pH 7.5, 100 mM NaCl, 10% Glycerol, 1 mM MgCl_2_, 0.001 mM GDP, 0.1 mM TCEP, and subsequently purified by size exclusion chromatography using a Superdex 200 10/300 GL (Cytiva) column and the same buffer as the eluent.

### Nanodisc assembly

Chloroform-solubilized lipids were mixed at the desired mol% ratios, vacuum-evaporated and resuspended at a final concentration of 25 mM in a 50 mM Tris-HCl pH 8, 2% SDS buffer. Nanodisc assembly then proceeded as described^54^. The GHSR was first folded in amphipol A8-35 from its SDS-unfolded state (see above) and A8-35 was exchanged to n-Dodecyl-β-D-Maltopyranoside (β-DDM)^33^. The GHSR was then bound onto a pre-equilibrated Ni-NTA Superflow resin, and incubated with 10 µM JMV3011, MSP1E3D1(-) and the lipid mixture at a 1:5:400 receptor:MSP:lipid ratio for 3 h. Polystyrene beads (BioBeads SM-2, BioRad) were added and incubated overnight at 4°C. The resin was washed with a 50 mM Tris-HCl pH 8.0, 150 mM NaCl, 1 µM JMV3011 buffer and the His-tagged receptor eluted with the same buffer supplemented with 200 mM imidazole. After extensive dialysis against 50 mM Tris-HCl pH 8, 150 mM NaCl, active receptor fractions were purified using affinity chromatography with the biotinylated JMV2959 immobilized on a streptavidin column. To this end, the receptor was loaded on the column, washed with a 50 mM Tris-HCl pH 8.0, 150 mM NaCl buffer and the ligand-competent protein eluted with the same buffer containing 1 mM JMV4183. Homogeneous fractions of GHSR-containing discs were finally obtained through a size-exclusion chromatography step on a S200 increase 10/300 GL column (Cytiva) using a 25 mM HEPES, 150 mM NaCl, 0.5 mM EDTA, pH 7.4 buffer as the elution buffer.

### GTP turnover assay

GTP turnover assay was conducted as described previously^21^. 200 nM GHSR in POPC:POPG (3:2) nanodiscs, with or without 10 μM MK0677, were incubated with 500 nM Gα_q_β_1_γ_2_ protein in a buffer 25 mM HEPES pH 7.4, 100 mM NaCl, 5 mM MgCl_2_ at 15 °C. GTP turnover was initiated by adding 5 μM GTP and 10 μM GDP, and the remaining GTP amount was assessed by measuring luminescence after 40 min incubation at 15 °C using the GTPase-Glo assay (Promega). The luminescence signal was normalized to the value obtained in the absence of receptor, which was set as 100%.

### Bimane labeling and fluorescence experiments

GHSR-containing nanodiscs with the specified lipid nanodisc composition were prepared using a Cys-min mutant of the GHSR with a single reactive cysteine at position 255^6.27^ ^21^. For both the receptor and β-arrestin1, the protein was labeled by incubating in the dark with a 10-fold molar excess of MB overnight at 4°C. Labeling was stopped by adding a 100-molar excess of L-Cys and incubating 1 h at 4 °C. Unreacted dye was removed by extensive dialysis. Labeling ratios were calculated from the absorption of the protein and that of the fluorophores at their corresponding maximum absorbance wavelengths (280 nm and 398 nm, respectively). Fluorescence measurements were performed on a Cary Eclipse fluorimeter (Varian) with a pulsed Xe-Lamp. For the GHSR, the receptor concentration was 5 μM, the excitation wavelength was set at 395 nm, and emission spectra were recorded between 420 and 600 nm with 0.1 nm intervals. For β-arrestin1, GHSR-containing nanodiscs (20 µM) were mixed with purified β-arrestin1 (2 µM) in a 25 mM HEPES, 50 mM NaCl, pH 7.4 buffer, and fluorescence measured after incubation for 2 h at 20 °C.

### GHSR:β-arrestin1 intermolecular FRET

GHSR nanodiscs and β-arrestin1 were first labeled by incubating them overnight at 4 °C in the dark with 1.0 equivalent of AlexaFluor 350 and AlexaFluor 488 maleimide (ThermoFisher), respectively. Labeling was terminated by adding a 100-fold excess of L-Cys. Unreacted dye was removed by extensive dialysis against 50 mM Tris-HCl pH 8.0, 150 mM NaCl. Labeling ratios were calculated from the absorption of the protein at 280 nm and that of the fluorophores at 346 nm (AlexaFluor 350) and 495 nm (AlexaFluor 488), using their known molar extinction coefficients. The GHSR was subsequently phosphorylated by incubating 1 µM GHSR-containing nanodiscs in the presence of the full agonist MK0677 (10 µM final concentration) with 1 µM human GRK5 in 50 mM Tris-HCl pH 8.0, 150 mM NaCl, containing 1 mM ATP and 1 mM MgCl_2_ overnight at RT. Protein phosphorylation was confirmed by SDS-PAGE followed by phosphoprotein-specific Pro-Q Diamond staining (ThermoFisher Scientific). The samples were extensively dialyzed in the same buffer as before to remove MK0677. 1 µM AF350-labeled phosphorylated GHSR nanodiscs were then incubated with 1 µM AF488-labeled β-arrestin1 in the presence or absence of 10 µM MK0677, in 50 mM Tris-HCl pH 8.0, 150 mM NaCl, containing 2 mM TCEP overnight at 4 °C. Fluorescence emission spectra were recorded on a Cary Eclipse spectrofluorometer (Varian) between 370 and 600 nm (1 nm intervals) with an excitation wavelength set at 345 nm.

### β-arrestin1 intramolecular FRET

The β-arrestin1 FRET assay was performed using β-arrestin1 labeled at position 12 with AlexaFluor 488 (donor) and at position 387 with AlexaFluor 647 (acceptor), using the conjugation maleimide protocol outlined in the previous section. Labeling ratios were calculated from the absorption of the protein at 280 nm and that of the fluorophores at 495 nm (AlexaFluor 488) and 650 nm (AlexaFluor 647), using their known molar extinction coefficients. FRET analysis was carried out for nanodiscs containing the GRK5-phosphorylated GHSR and the indicated lipid composition by using a 2:1 GHSR-to-arrestin molar ratio. FRET measurements were performed using a Cary Eclipse spectrofluorometer (Varian), with an excitation wavelength set at 488 nm and emission recorded between 500 and 750 nm with 1 nm intervals.

### DNS fluorescence

The cysteine-free mutant of β-arrestin1 with a single reactive cysteine at position 341 was incubated in the dark with a 5-fold molar excess of DNS-maleimide (Sigma Aldrich) overnight at 4 °C. Excess fluorophore was removed through a SEC purification step on an Superdex 200 increase 10/300 GL column (Cytiva) using a 20 mM HEPES pH 7.5, 200 mM NaCl, 10% glycerol as the eluent. Labeling ratios were estimated from the absorbance intensities of the protein and DNS at their respective maximum emission wavelengths (280 nm and 337 nm, respectively). Fluorescence emission curves were measured with a Fluoromax-4 spectrofluorometer (Horiba Scientific), with an excitation wavelength set at 337 nm and the emission wavelengths between 400 nm and 700 nm with 1 nm intervals.

### Laurdan fluorescence

Receptor-free nanodiscs containing increasing mol% in cholesterol were prepared as described above and mixed with Laurdan at a mol% ratio of 500:1. The mixture was incubated in the dark at 25 °C for 1 h and the resulting nanodiscs purified by size exclusion chromatography using a Superdex 200 increase 10/300 GL column (Cytiva) with a 25 mM Na-HEPES, 150 mM NaCl, 0.5 mM EDTA buffer as the eluent. Fluorescence emission curves were measured with a Fluoromax-4 spectrofluorometer (Horiba Scientific), with an excitation wavelength set at 366 nm and the emission wavelengths between 400 nm and 600 nm with 1 nm intervals. The resulting emission spectra were normalized to the maximum emission intensity and generalized polarization (GP) was calculated from GP = (I_440_-I_490_)/I_440_ + I_490_), where I_440_ and I_490_ are the emission intensities at 440 nm and 490 nm, respectively.

### 7-H4MC fluorescence measurements

Ghrelin(1-10) derivatives with a 7H4MC moiety at position 4 were synthesized as described^27^. This peptide was incubated for 2 h in a 25 mM Na-HEPES, 150 mM NaCl, 0.5 mM EDTA buffer in the presence of GHSR-containing nanodiscs at a 1:10 ghrelin-to-GHSR molar ratio (GHSR concentration: 5 µM), in the absence or in the presence of a 10-fold excess (with regard to the receptor) of β-arrestin1. Fluorescence spectra were recorded with a FluoroMax-4 spectrofluorometer (Horiba Scientific). The emission spectra after excitation at 320 nm were recorded between 375 and 600 nm with 1 nm intervals. The normalized emission intensity was fitted by means of nonlinear least-square procedure to the sum of peak function^28^. The hydration parameter H was calculated as the sum of the contributions of the anionic and tautomer forms.

### Ligand binding assays

Competition ligand-binding assays were performed using fluorescence energy transfer with a purified receptor labeled at its N-terminus with Lumi-4 Tb NHS and a dy647-labeled ghrelin peptide^29^. Increasing concentrations in the competing compound were added to a receptor:ghrelin peptide mixture (100 nM concentration range). After a 60 min incubation at 15 °C, fluorescence emission spectra were recorded at the same temperature between 400 and 600 nm (Cary Eclipse spectrofluorimeter, Varian) with excitation at 346 or 488 nm.

### Statistics and reproducibility

Statistical analyses were carried out with GraphPad Prism 10. Data are presented as mean ± SD of at least three independent experiments, as specified in the figure legends. All datasets were systematically considered.

### Illustrations

All schematic representations were created in https://BioRender.com.

### Reporting summary

Further information on research design is available in the Nature Portfolio Reporting Summary linked to this article.

## Supplementary information

Supplementary Information

Supplementary data

Description of Additional Supplementary Files

reporting summary

## Data Availability

The authors declare that the data supporting the findings of this study are available within the paper and its Supplementary Data. Should any raw data files be needed in another format they are available from the corresponding authors upon reasonable request.
