# Supplementary material for: Membrane cholesterol modulates engagement of β-arrestin with the ghrelin receptor
Source: Commun Biol. 2026 Apr 1;9:782. doi: 10.1038/s42003-026-09889-0 (PMC13250087; doi:10.1038/s42003-026-09889-0)
Supplement: Supplementary file 1 — Supplementary Information [file 42003_2026_9889_MOESM1_ESM.pdf]

Supporting Information for

Membrane cholesterol modulates core engagement of β-arrestin with the ghrelin receptor

Ludovic Berto[a,1], Pauline Henri[a,1], Marjorie Damian[a], Sonia Cantel[a], Jean-Alain Fehrentz[a], Nathalie Sibille[b], Michela Di Michele[a,2] and Jean-Louis Banères[a,2]

[a]Institut des Biomolécules Max Mousseron (IBMM), CNRS, Université de Montpellier, ENSCM, Montpellier, France.
[b]Centre de Biologie Structurale (CBS), Université de Montpellier, INSERM, CNRS, Montpellier, France.

[1]L.B. and P.H. contributed equally to this work.

[2]to whom correspondence may be addressed. Email: michela.di-michele@enscm.fr, jean-louis.baneres@umontpellier.fr

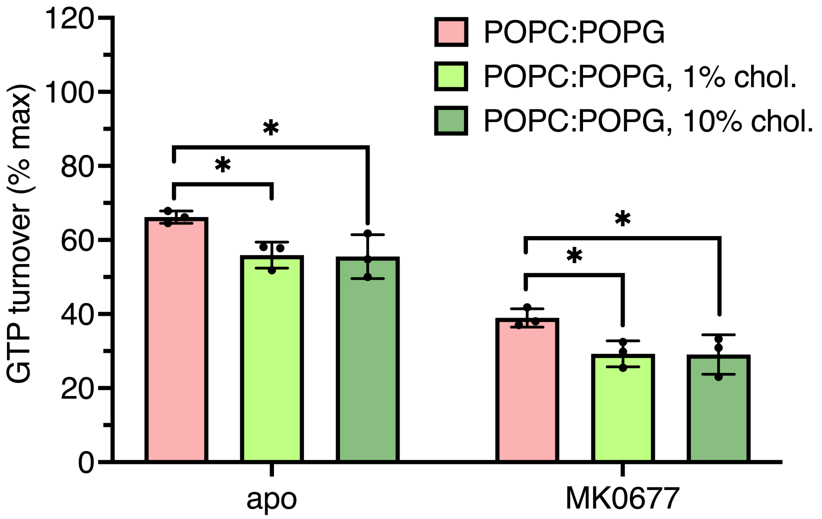

Figure S1. Effect of cholesterol on receptor-catalyzed Gq activation. GTP turnover for Gq catalyzed by the GHSR in POPC:POPG nanodiscs, or in the same nanodiscs containing either 1% or 10% cholesterol in the absence or presence of 10 µM MK0677. The signal was normalized to that obtained for the G protein in the absence of receptor. Data is mean ± SD of three replicates per group, with *p≤ 0.05, by one-way ANOVA test with Bonferroni post-test.

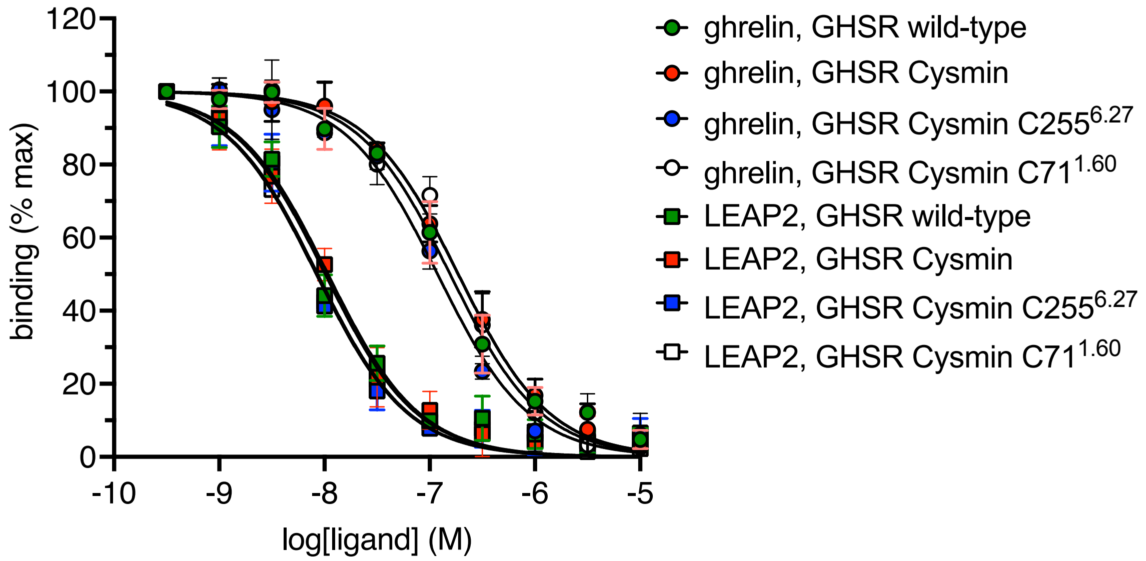

Figure S2. Effect of the mutations on the ligand-binding of the GHSR. FRET-monitored competition assay between the labeled ghrelin (1-17) peptide labeled with dy-647 at its C-terminus and either ghrelin (1-17) or LEAP2 (1-12) for binding the wild-type GHSR, its minimal cysteine mutant (GHSR Cysmin), or the same minimal cysteine mutant including a unique reactive cysteine at position 71$^{1.60}$ or 255$^{6.27}$. In all cases, the receptor was labeled with Lumi4-Tb at its N-terminus. Data are presented as the mean ± SD from three experiments.

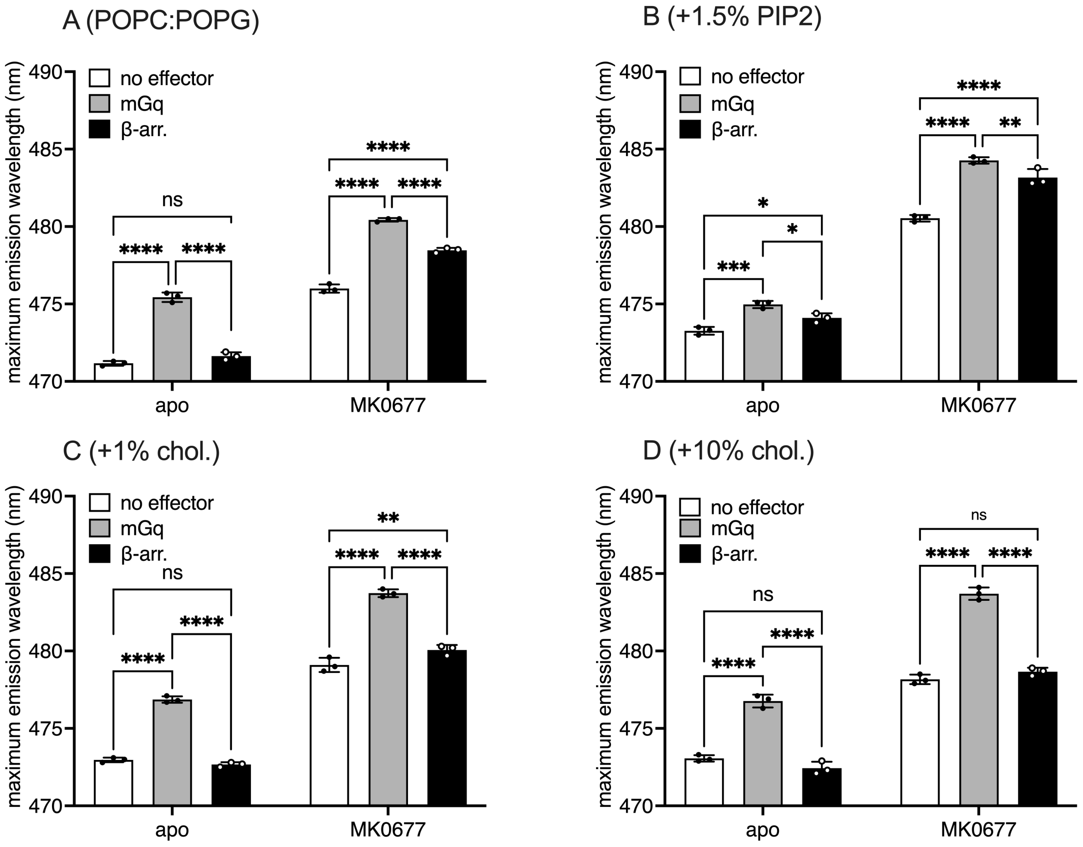

Figure S3. Modulation by PIP2 or cholesterol of the effect of the effectors on the GHSR conformational equilibria. MB emission maximum wavelength for the GHSR in nanodisc composed of POPC:POPG (A), POPC:POPG, 1.5% PIP2 (B), POPC:POPG, 1% cholesterol (C) or POPC:POPG, 10% cholesterol (D), in the absence of effector or in the presence of either mGq or ΔCter β-arrestin1. Data are mean ± SD of three replicates per group. ns not significant, *p≤ 0.05, ** P ≤ 0.01, ***p ≤ 0.001, and ****p ≤ 0.0001, by one-way ANOVA test with Bonferroni post-test. Data are from Figure 1 and Figure 2.

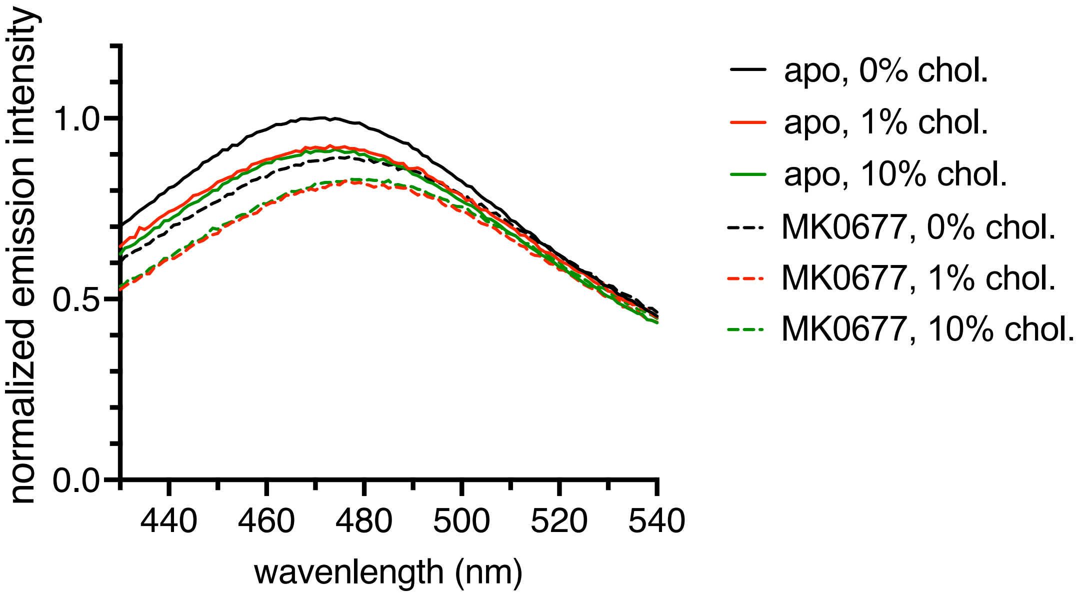

Figure S4. Effect of cholesterol on the GHSR conformational equilibria. MB emission spectra of the GHSR in the absence or presence of either 1% or 10% cholesterol in the nanodiscs, in the absence of ligand or in the presence of 10 µM MK0677. The spectra are representative of one of three experiments and were normalized to the maximum emission intensity value measured with the apo receptor in the absence of cholesterol.

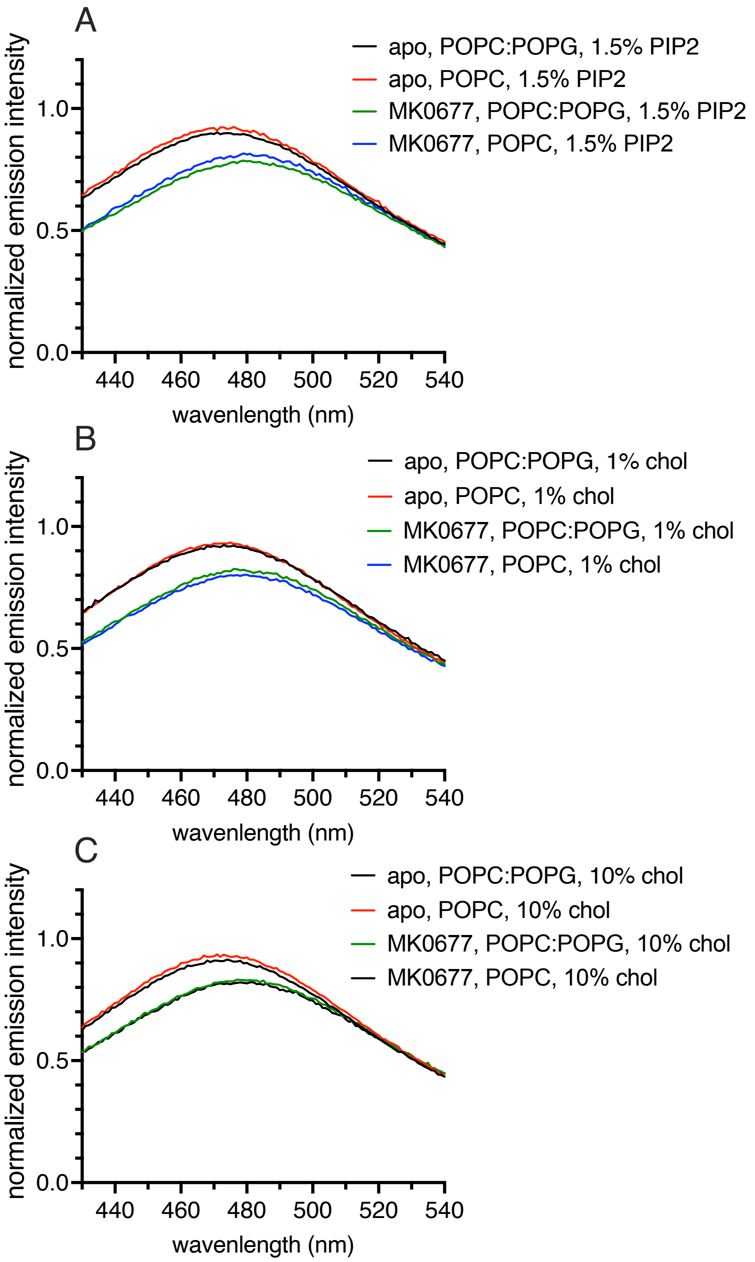

Figure S5. POPG does not impact of GHSR conformational equilibria. MB emission spectra of the GHSR assembled into either POPC-only or POPC:POPG nanodiscs, in the absence of ligand or in the presence of 10 µM MK0677, and in the presence of either 1.5% PIP2 (A), 1% cholesterol (B) or 10% cholesterol (C). The spectra are representative of one of three experiments and were normalized to the maximum emission intensity value measured with the apo receptor in the absence of PIP2 and cholesterol.

.

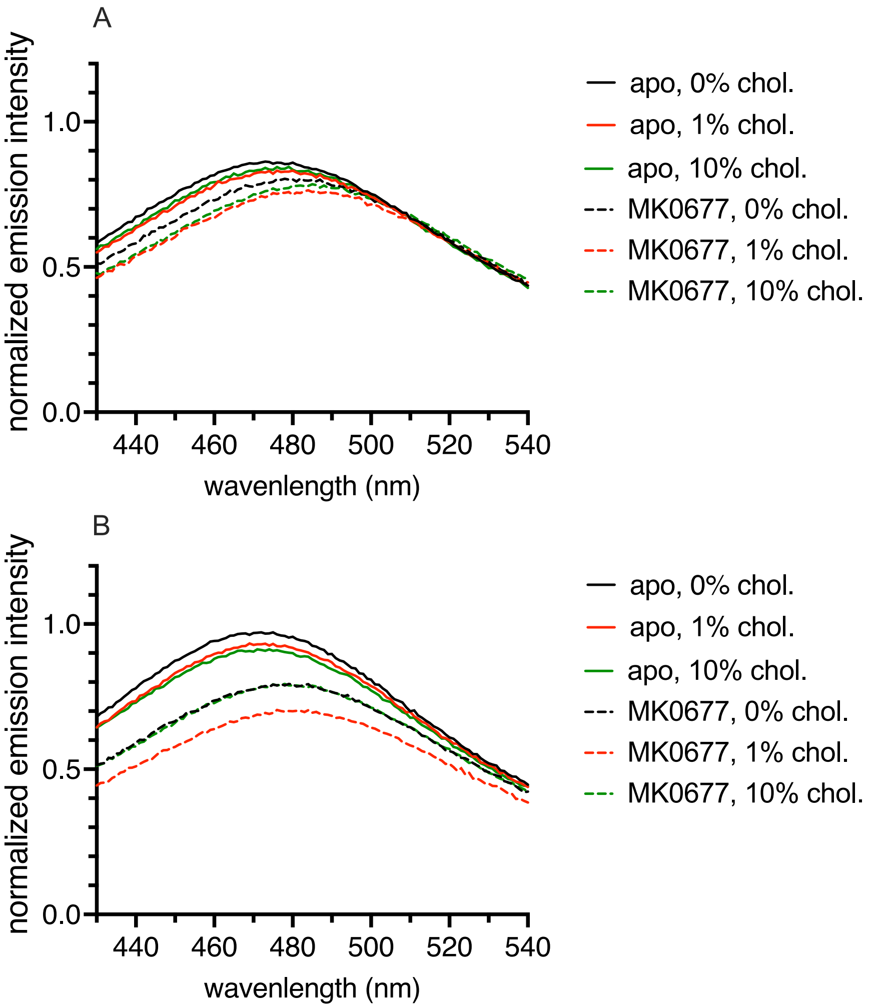

Figure S6. Effect of signaling proteins and cholesterol on the GHSR conformational equilibria. (A) MB emission spectra in the presence of mGq, in the absence or in the presence of 10 μM MK0677, and in the presence of either 11% or 10% cholesterol in the nanodiscs. (B) Same as in (A) but in the presence of ΔCter β-arrestin1 instead of mGq. The spectra are representative of one of three experiments and were normalized to the maximum emission intensity value measured with the apo receptor in the absence of cholesterol.

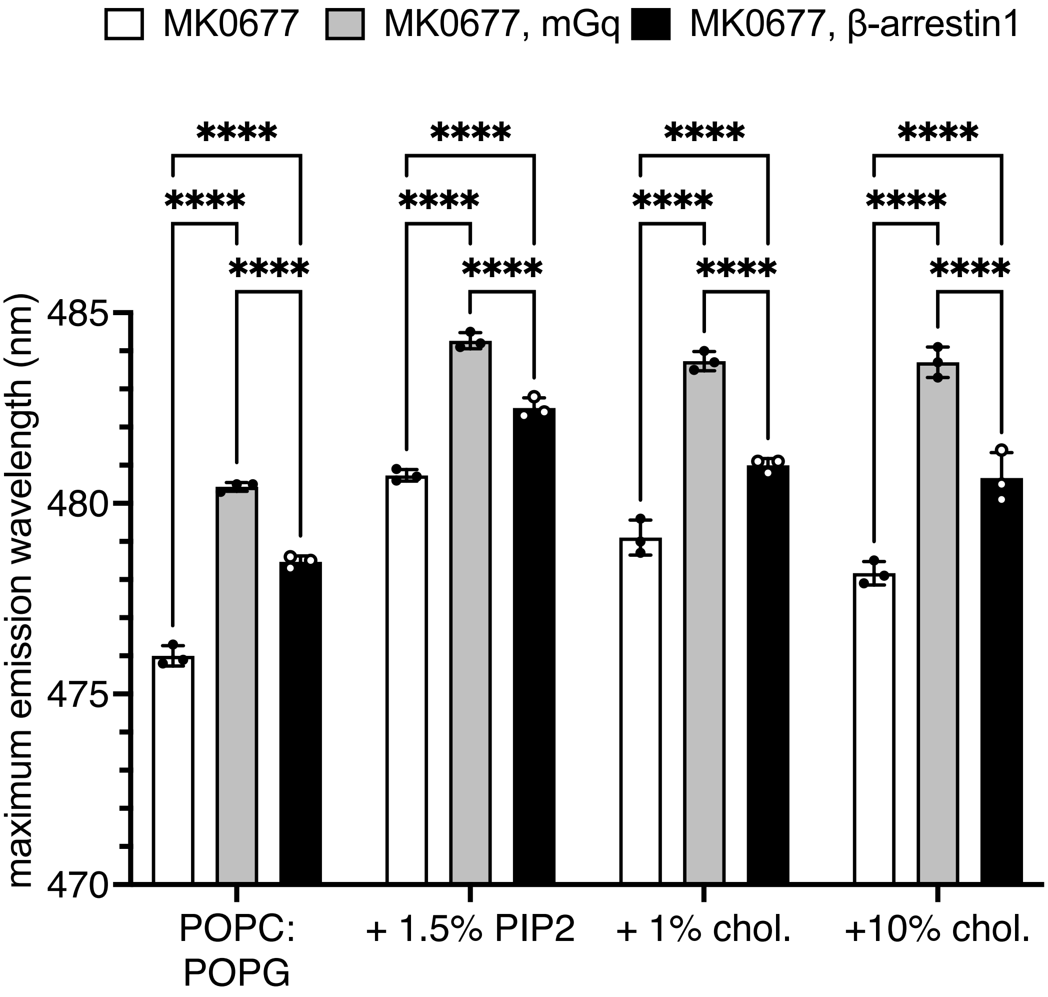

Figure S7. Relative effect of mGq and ΔCter β-arrestin1 on the GHSR conformational equilibrium. Maximum emission wavelength of MB in the absence of effector or in the presence of either mGq or ΔCter β-arrestin1, in the presence of 10 µM MK0677, and in the presence of either 1.5% PIP2, 1% or 10% cholesterol in the nanodiscs. Data are mean ± SD of three replicates per group. ****p ≤ 0.0001, by one-way ANOVA test with Bonferroni post-test. Data are from Figure 2.

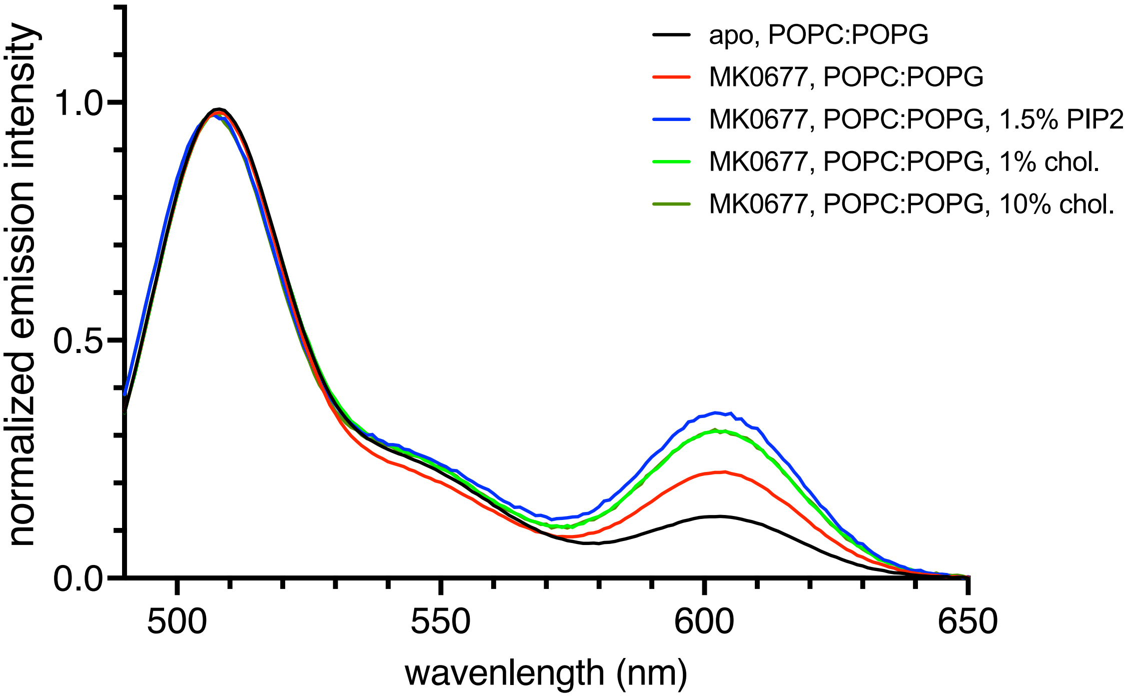

Figure S8. Effect of PIP2 or cholesterol on mini-G protein recruitment to the GHSR. Normalized emission spectra of the AlexaFluor 568-labeled GHSR in POPC:POPG nanodiscs in the presence of GFP-mGq protein (receptor-to-mGq molar ratio 1:2.5), in the absence or presence of 10 µM MK0677, and in the absence or presence of either 1.5% PIP2, 1% cholesterol or 10% cholesterol in the nanodiscs. Spectra are normalized to the donor intensity within a given experiment, and are shown for one representative of three experiments.

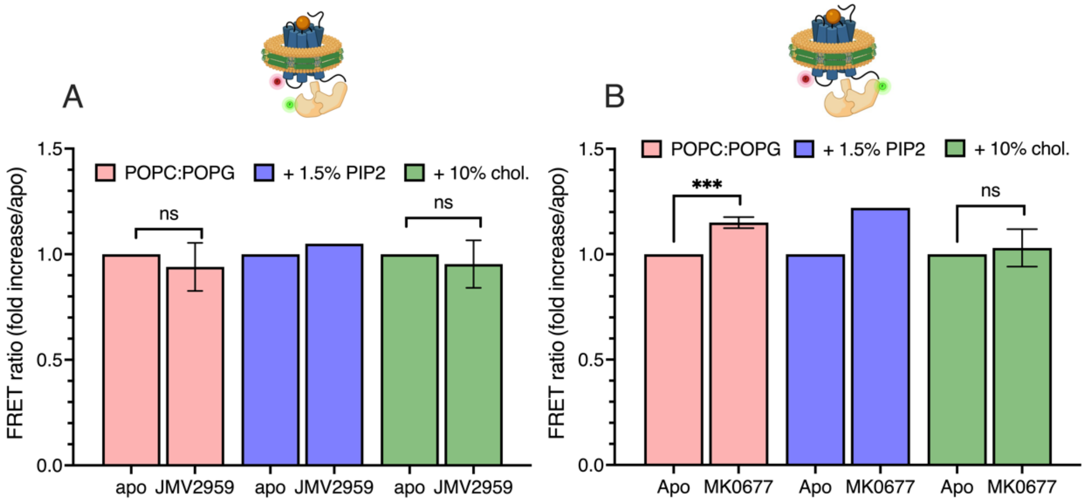

Figure S9. Effect of JMV2959 binding and labeling position on the GHSR:β-arrestin1 interaction. (A) Changes in the FRET ratio observed with the GHSR labeled with AlexaFluor 350 and β-arrestin1 labeled with AlexaFluor 488 on C167 in the absence of ligand or in the presence of 10 μM JMV2959, and in the absence or presence of 1.5% PIP2 or 10% cholesterol in the nanodiscs. (B) Changes in the FRET ratio observed with the GHSR labeled with AlexaFluor 350 at position 71 and β-arrestin1 labeled with AlexaFluor 488 on C191 in the absence of ligand or in the presence of 10 μM MK0677, and in the absence or presence of 1.5% PIP2 or 10% cholesterol in the nanodiscs. ns: not significant, ***$p \leq 0.001$, by non-parametric unpaired t test.

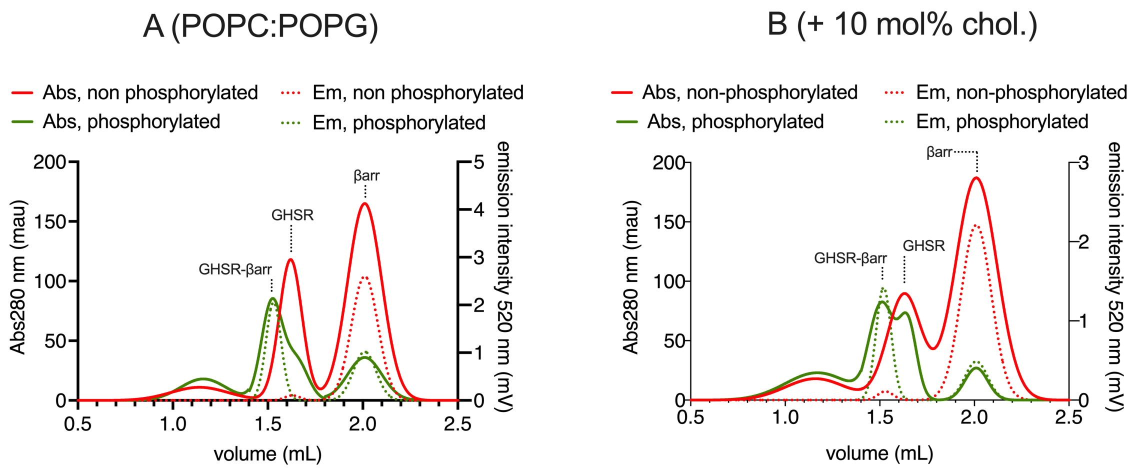

Figure S10. F-SEC analysis of the GHSR:β-arrestin1 complex. Size exclusion chromatography profile of a 1:5 mixture of the non-phosphorylated or GRK5-phosphorylated GHSR in nanodiscs devoid (A) or containing (B) 10% cholesterol and β-arrestin1 labeled with Alexa Fluor 488 at position 12. Chromatography was run on a S200 increase 5/150 column (Cytiva) using a 25 mM Na-HEPES, 200 mM NaCl, 1 µM MK0677 buffer as the eluent. The elution profile was monitored through the measure of the absorbance at 280 nm and the emission of Alexa Fluor 488 at 520 nm (λexc: 499 nm).

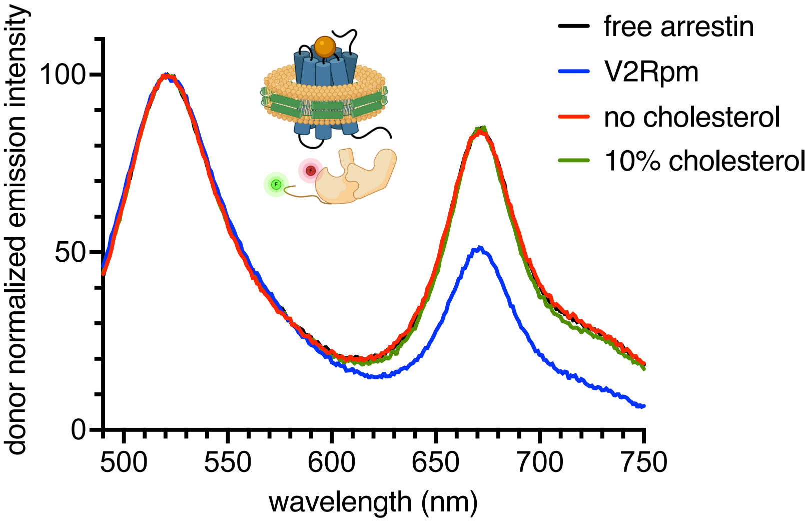

Figure S11. Effect of GHSR phosphorylation on β-arrestin1 coupling and activation. Emission spectra of wild-type β-arrestin1 labeled with AlexaFluor 488 and AlexaFluor 647 in the free state, in the presence of 50 µM V2R C-tail phosphomimetic peptide, or in the presence of nanodiscs containing unphosphorylated GHSR, in the absence or in the presence of 10% cholesterol in the nanodiscs. Spectra are normalized to the maximum emission of the donor of each spectrum and are representative of one of three experiments.

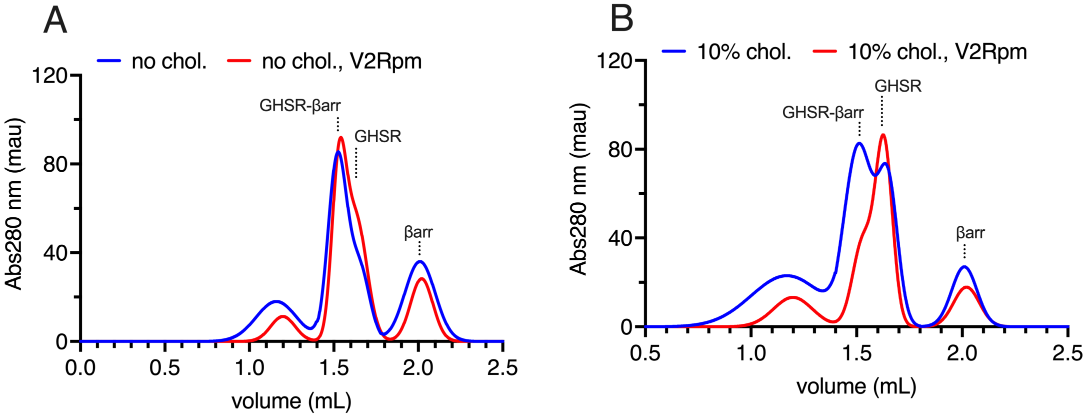

Figure S12. Stability of the GHSR:β-arrestin1 complex. Size exclusion chromatography profile of a 1:5 mixture of GRK5-phosphorylated GHSR in nanodiscs devoid (A) or containing (B) 10% cholesterol and β-arrestin1, in the absence or in the presence of a 50-fold excess in the V2R phosphomimetic peptide. Chromatography was run on a S200 increase 5/150 column (Cytiva) using a 25 mM Na-HEPES, 200 mM NaCl, 1 µM MK0677 buffer as the eluent. The elution profile was monitored through the measure of the absorbance at 280 nm.

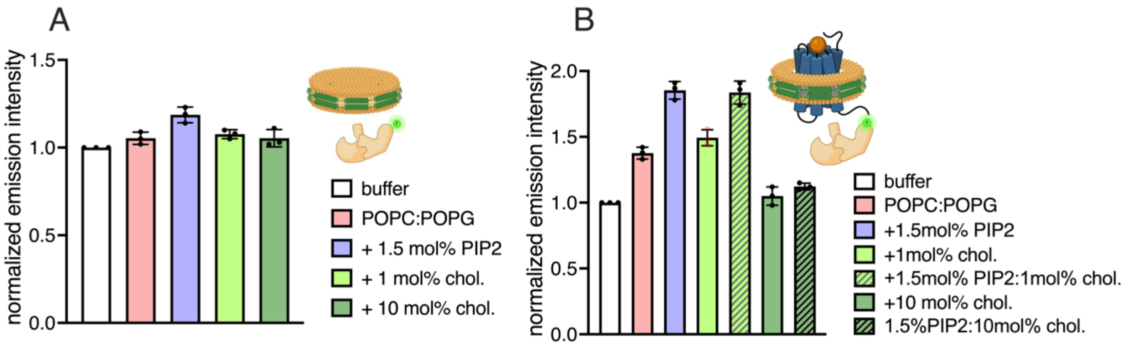

Figure S13. Cholesterol affects β-arrestin1 interaction with the bilayer. (A) DNS normalized maximum emission intensity of β-arrestin1 labeled on C341 free in solution or in empty nanodiscs of different lipid composition. (B) same experiment with GHSR-containing nanodiscs. In (B), data in POPC:POPG, POPC:POPG:1.5% PIP2, POPC:POPG:1% cholesterol and POPC:POPG:10% cholesterol are from Figure 5. Values are mean ± SD of three replicates per group.

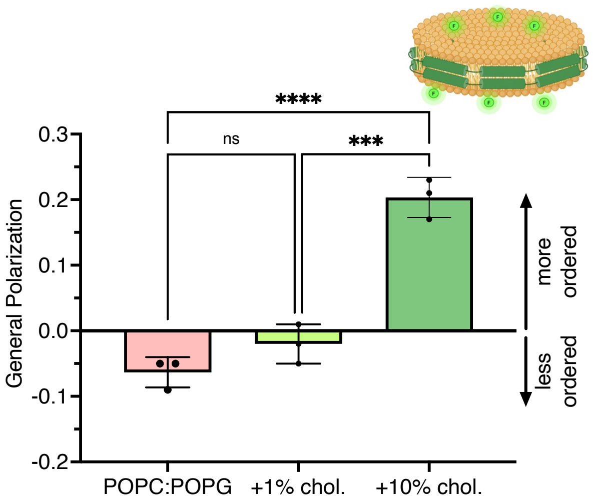

Figure S14. Effect of cholesterol on membrane lipid order in nanodiscs. Laurdan general polarization measured for receptor-free POPC:POPG nanodiscs at increasing amounts of cholesterol.
