# Supplementary material for: Membrane cholesterol modulates engagement of β-arrestin with the ghrelin receptor
Source: Commun Biol. 2026 Apr 1;9:782. doi: 10.1038/s42003-026-09889-0 (PMC13250087; doi:10.1038/s42003-026-09889-0)
Supplement: Supplementary file 3 — Description of Additional Supplementary Files [file 42003_2026_9889_MOESM3_ESM.docx]

Description of Additional Supplementary File

File name: Supplementary Data
Description: All numerical data is provided as sperate tabs in an Excel file.
